# Fused Omics Data Models Reveal Gut Microbiome Signatures Specific of Inactive Stage of Juvenile Idiopathic Arthritis in Pediatric Patients

**DOI:** 10.3390/microorganisms8101540

**Published:** 2020-10-06

**Authors:** Pamela Vernocchi, Federico Marini, Giorgio Capuani, Alberta Tomassini, Giorgia Conta, Federica Del Chierico, Clara Malattia, Fabrizio De Benedetti, Alberto Martini, Bruno Dallapiccola, E. H. Pieter van Dijkhuizen, Alfredo Miccheli, Lorenza Putignani

**Affiliations:** 1 Area of Genetics and Rare Diseases, Unit of Human Microbiome, Bambino Gesù Children’s Hospital, IRCCS, 00165 Rome, Italy; pamela.vernocchi@opbg.net (P.V.); federica.delchierico@opbg.net (F.D.C.); 2 Department of Chemistry, Sapienza University of Rome, 00185 Rome, Italy; federico.marini@uniroma1.it (F.M.); giorgio.capuani@uniroma1.it (G.C.); alberta.tomassini@uniroma1.it (A.T.); giorgia.conta@uniroma1.it (G.C.); 3 NMR-Based Metabolomics Laboratory, Sapienza University of Rome, 00185 Rome, Italy; alfredo.miccheli@uniroma1.it; 4 Seconda Divisione di Pediatria, Istituto Giannina Gaslini and Università degli Studi di Genoa, 16147 Genoa, Italy; claramalattia@gaslini.org (C.M.); albertomartini@gaslini.org (A.M.); e.h.p.dijkhuizen@umcutrecht.nl (E.H.P.v.D.); 5 Department of Pediatric Subspecialties, Division of Nephrology, Bambino Gesù Children’s Hospital, IRCCS, 00165 Rome, Italy; fabrizio.debenedetti@opbg.net; 6 Scientific Directorate, Bambino Gesù Children’s Hospital, IRCCS, 00165 Rome, Italy; bruno.dallapiccola@opbg.net; 7 Department of Pediatrics, University Medical Center Utrecht, Wilhelmina Children’s Hospital, 3584 EA Utrecht, The Netherlands; 8 Department of Environmental Biology, Sapienza University of Rome, 00185 Rome, Italy; 9 Unit of Parasitology and Area of Genetics and Rare Diseases, Unit of Human Microbiome, Department of Laboratories, Bambino Gesù Children’s Hospital, IRCCS, 00165 Rome, Italy

**Keywords:** gut microbiome, gut metabolome, volatile-organic compounds, Non-VOCs, operational taxonomic units, fused omics data, microbiomics

## Abstract

Juvenile idiopathic arthritis (JIA) is the most common rheumatic disease in children. Herein, we evaluated the relationship between the gut microbiome (GM) and disease phenotype by an integrated omics fused approach. In a multicenter, observational cohort study, stools from Italian JIA patients were collected at baseline, active, and inactive disease stages, and their GM compared to healthy controls (CTRLs). The microbiota metabolome was analyzed to detect volatile- and non-volatile organic compounds (VOCs); the data were fused with operational taxonomic units (OTUs) from 16S RNA targeted-metagenomics and classified by chemometric models. Non-VOCs did not characterize JIA patients nor JIA activity stages compared to CTRLs. The core of VOCs, (Ethanol, Methyl-isobutyl-ketone, 2,6-Dimethyl-4-heptanone and Phenol) characterized patients at baseline and inactive disease stages, while the OTUs represented by Ruminococcaceae, Lachnospiraceae and Clostridiacea discriminated between JIA inactive stage and CTRLs. No differences were highlighted amongst JIA activity stages. Finally, the fused data discriminated inactive and baseline stages versus CTRLs, based on the contribution of the invariant core of VOCs while Ruminococcaceae concurred for the inactive stage versus CTRLs comparison. In conclusion, the GM signatures enabled to distinguish the inactive disease stage from CTRLs.

## 1. Introduction

In recent years, many evidences have highlighted that alterations in the gut microbiome (GM) were frequently associated to the development of local and systemic inflammatory and autoimmune diseases [1], and thus, the role of GM in the pathogenesis of juvenile idiopathic arthritis (JIA) has been also hypothesized [2]. In a recent multicenter observational cohort study, a GM dysbiosis for JIA patients was detected, in terms of richness and compositional deviation from age-matched healthy controls (CTRLs) [3]. Particularly, the study of van Dijkhuizen et al. [3] included a large cohort of treated-naive JIA Dutch and Italian patients in longitudinal follow up. This experimental design, which allowed us to compare the GM amongst patients versus CTRLs, revealed that age, geographic origin and disease status appeared to be determinant factors for the GM signature, regardless of the disease activity stage (i.e., baseline, inactive, active) and inflammation markers. In addition, a recent re-evaluation of previously published data derived from USA [4], Finland [5] and Indian [6] cohorts confirmed that the GM signature of JIA patients was different from CTRLs, on the basis of their geographical origin [7]. Moreover, training mathematical models built on a single cohort was unable to differentiate JIA patients from CTRLs [7]. Hence, while the JIA disease status is ascertained to be associated to a dysbiosis, the role of GM composition in the pathogenesis of the disease must be still deeply investigated, particularly for the aspects depending on geographical variability. The present study highlights a progression of a previous clinical study [3]; in particular, a well-defined Italian cohort of JIA patients at baseline, inactive and persistent stage conditions compared to CTRLs was studied to find possible associations amongst composition (operational taxonomic units OTUs at bacterial family level) and content of volatile organic compound (VOCs) and low weight molecules (Non-VOCs) in the stools. The multi-omic approach, achieved by the integration of different metabolomics and targeted-metagenomics data, might provide different profiles to better highlight the role of GM in the JIA disease.

## 2. Materials and Methods

### Patient Enrollment and Omics Procedures

This observational prospective cohort study was conducted in Italy at the Ospedale Pediatrico Giannina Gaslini (OPGG) of Genoa and at the Ospedale Pediatrico Bambino Gesù (OPBG) of Rome, Italy. The consecutive 60 patients’ cohort, 46 from OPBG and 14 from OPGG, respectively, was recruited between October 2013 and December 2015 with a JIA diagnosis, according to International League Against Rheumatism (ILAR) criteria [8].

Patients, 16 males and 44 females, aged 1.7 to 16.6 years (average age 7.0 years SD ± 4.11), were aged-matched against a cohort of 25 CTRLs subjects, screened during a survey on GM programming at the OPBG Human Microbiome Unit (Table 1). Moreover, clinical data including the JADAS- 71 [9], CRP, ESR and the use of NSAIDs were collected (Table 1), as well as information on dietary habits and prebiotic, probiotic administration (Table S1). At the follow up visits, disease activity status was defined according to the Wallace criteria for inactive disease [10]. A previous study included more detailed information on the same patient’s cohort [3].

The study was approved by the OPBG ethics committees (Code 615/2013) on 6 May 2013 and was conducted in accordance with the Principles of Good Clinical Practice and the Declaration of Helsinki. Written informed consent was obtained from all participants.

Eighty-five fecal samples were collected at the reference node of the Biobanking and Biomolecular Resources Research Infrastructure of Italy (BBMRI) for human microbiome of the OPBG Human Microbiome Unit and stored at −80 °C until further meta-omics processing.

The 16S targeted-metagenomics data of the GM profiling, discussed in the previous study [3], were harmonized and combined for omics data integration to metabolomics data obtained by both solid phase micro-extraction coupled with gas-chromatography mass spectrometry (SPME/GC-MS) (Table S2) and nuclear magnetic resonance (NMR) spectroscopy (Table S3), obtained as described in the previous studies [11,12].

**Figure 1:**
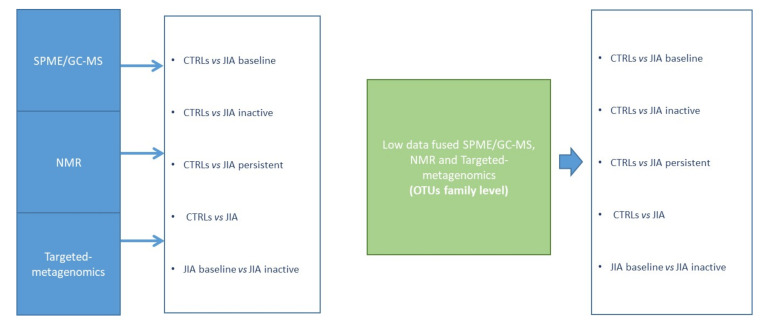
Strategy for the multivariate analysis of targeted-metagenomics, gas-chromatography mass spectrometry SPME/GC-MS and nuclear magnetic resonance NMR based metabolomics data and low data fused (integrated platforms). Classification models, based on Partial least squares discriminant analysis(PLS-DA) in double cross-validation (DCV), were at first established considering the results for each omic platform and, in a second stage, according to a low-level data fusion strategy (targeted-metagenomics, SPME/GC-MS and NMR).

To integrate the multidimensional data, univariate and multivariate analyses were performed by MATLAB toolbox or by in-house written software. The OTUs and SPME/GC-MS raw data were reduced in order to obtain matrices considering only OTUs and metabolites detected in at least 70% of the entire dataset, including both JIA and CTRLs subjects. On the contrary, NMR raw data matrix did not need any reduction. Classification models were built according to Vernocchi et al. [13] and optimized for this specific application (Figure 1). Moreover, the partial least square-discriminant analysis (PLS-DA) was performed according to Szymańska et al. [14]; in particular, validation was carried out by means of a combination of double cross-validation (DCV) and permutation tests. Following DCV procedure, for each variable the geometric average was considered, and the non-parametric statistics rank product (RP) index was applied [15]. Low values of RP indicated variables highly contributing to the model and, accordingly, considered as candidate biomarkers.

## 3. Results

### 3.1. Patients

Sixty JIA pediatric patients, (average age 7.0 years, SD ± 4.11), compared to 25 healthy age-paired subjects, were recruited in a previous study [3], to evaluate their microbiota signatures by metabolomics profiling and data fusion with microbiota composition. In according to the Juvenile Arthritis Disease Activity Score in 71 joints (JADAS-71) [9,16], the disease activity varied from moderate (mean value: 15.95) for baseline, to low (mean values 0.34) for inactive and persistent (mean values 7.19) disease stages, respectively. The mean values of C-reactive protein (CRP) showed low levels in the serum ranging from 0.24 to 1.38 mg/L. The erythrocyte sedimentation rate (ESR) resulted higher at baseline (mean value 24.6 mm/hour), followed by ESR values at persistent (mean value 18.4 mm/h) and inactive disease stage (mean value 11.1 mm/h). Nonsteroidal anti-inflammatory drugs (NSAIDs) were mainly administered to patients at baseline (15/20), followed by patients with persistent (7/21) and inactive disease (3/19). No patients used any biologicals or synthetic antirheumatic drugs at baseline, whereas most patients were on methotrexate (MTX) in inactive (14/19) and during persistent (12/21) disease stages. Some patients used biological drugs (Table 1).

### 3.2. Omics Data and Fused Model Analysis

Different classification algorithms were created in order to achieve a functional model of the GM in JIA. Firstly, the analysis was conducted separately on the different data resulting from single platforms and generated variables: (i) targeted-metagenomics and OTUs; (ii) SPME/GC-MS and VOCs; (iii) NMR and Non-VOCs (Figure 1).

The PLS-DA models were created on single and low-level fused data generated from VOCs, OTUs, and Non-VOCs.

Focusing on VOCs, the average correct classification rate (CCR3) referring to CTRLs versus JIA patients (total variables) comparison ranged around 77% for each paired CTRLs-disease stage. Particularly, registered percentages were: 78.5 ± 2.6, CTRLs-JIA Baseline; 75.9 ± 4.9, CTRLs-JIA Inactive; 77.5 ± 3.2, CTRLs-JIA Persistent. Overall, CTRLs versus entire JIA phenotypes were classified by averaged CCR 3 62.9 ± 2.7% (Table 2).

Despite the partial CCR for each JIA disease stage was lower (CCR1 average around 55%) with respect to CTRLs group (CCR2 average around 95%), the CCR3 for the paired CTRLs-JIA disease stages represented a high value of classification. However, its value was principally due to CTRLs, hence characterizing this model as having a low predictive ability to identify JIA disease stage groups (Table 2). Nonetheless, an invariant core of metabolites such as Methyl-isobutyl-ketone, Ethanol, 2,6-Dimethyl-4-heptanone and Phenol were shared by the metabolomics profiles of patients at baseline and inactive disease stages, therefore resulting overrepresented in the patients’ profiles. The Methyl-isobutyl-ketone and the 1-Hexanol represented the highest and the lowest represented metabolite, respectively, in the JIA PLS-DA model (Table 3).

Moreover, OTUs (Table 4 and Table 5) and Non-VOCs (Table 6) variables, evidenced values of CCRn that failed in the attempt to classify all groups of variables, including JIA, CTRLs and JIA at different disease stage.

Concerning OTUs, the calculated values for each JIA disease stage (CCR1 average around 60%) and CTRLs (CCR2 average around 61%) resulted in a low prediction model (Table 4). Particularly, registered average percentages were: 53.3 ± 4.2, CTRLs-JIA Baseline; 69.4 ± 4.0, CTRLs-JIA Inactive; 58.1 ± 4.7, CTRLs-JIA Persistent. Therefore, the comparison between CTRLs and JIA Inactive stage (Table 4) was characterized by the highest CCRn value. The value was achieved by the concurrent contribution of the OTUs at family level, Ruminococcaceae, Lachnospiraceae, Clostridiaceae (Table 5).

Non-VOCs variables (Table 6) showed average and single CCRn values around 40–60%; hence the alone NMR platform data were considered not reliable to perform a group classification validation for both CTRLs, JIA patients and single JIA-disease stages.

Moving to the low-level fused data, the total classification ability of the model ranged from 57.6 to 81.2%. In particular, the grouping between JIA disease stage and CTRLs was classified by CCR 3 score whose average ranged from 68.1 to 72.8% (Table 7). For the comparison CTRLs/JIA ALL, the CCR 3 average value was the lowest value compared to the other CCR3, while, within disease phenotypes, the model recognized both JIA baseline (CCR3 71.6 ± 4.9) and JIA inactive stages (CCR3 72.8 ± 5.1) from CTRLs, as reported in Table 7.

**Figure 2:**
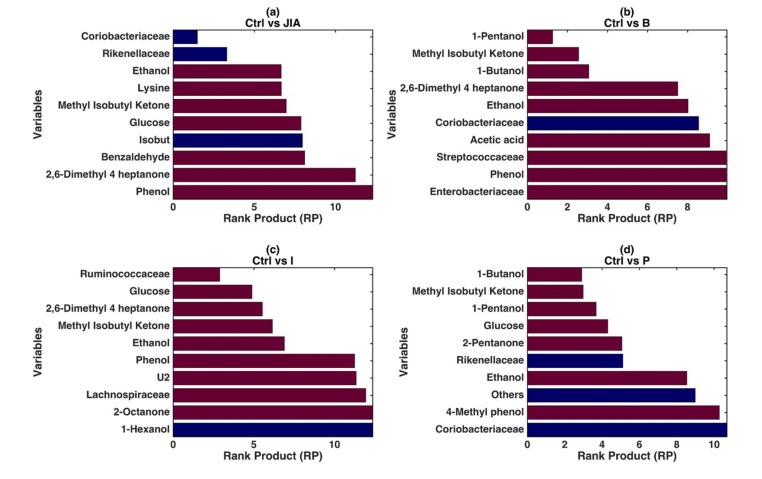
PLS-DA modeling on the low-level fused data. Graphical illustration of the variables significantly contributing to the definition of the single models based on the values of RP resulting from the repeated DCV procedure. The four different panels show the RP values for the models: (**a**) CTRLs vs. JIA; (**b**) CTRLs vs. JIA Baseline; (**c**) CTRLs vs. JIA Inactive; (**d**) CTRLs vs. JIA Persistent. In each panel, the color of the bars indicates whether, on average, the variable is higher (red) or lower (blue) in the specific JIA disease stage with respect to the CTRLs.

In the fused model, the variables OTUs and metabolites, which could be identified as significant and predictive compared to the CTRLs, were defined by the RP obtained by DCV procedure (Figure 2). Overall, the models built on the JIA patients at baseline, active and persistent disease stage identified high levels of alcohols, such as 1-Butanol and 1-Pentanol. Moreover, low abundance of Coriobacteriaceae represented an important variable for the discrimination from CTRLs of JIA Baseline and with less representativity of persistent disease stages’ children (Figure 2a).

The VOCs Ethanol, Methyl-isobutyl-ketone, 2,6-Dimethyl-4-heptanone and Phenol represented the metabolites discriminating JIA patients at both baseline and inactive stage from CTRLs in the PLS-DA (Figure 2b,c). These VOCs at JIA Baseline showed RP values ranging from 3 to 10, in the model discriminating CTRLs vs. JIA Baseline while in CTRLs vs. JIA Inactive RP values ranged from 6 to 12, identifying these molecules as highly contributing to the models, hence representing potential biomarkers (Figure 2b,c) of the disease stages. Streptococcaceae (RP: > 10) and Enterobacteriaceae (RP: > 10) were associated with JIA Baseline, despite low model values (Figure 2b). On the other site, Ruminococcaceae (RP: 4) well represented JIA Inactive stage, with also the low scored contribution of Lachnospiraceae (RP: 12.5) (Figure 2c).

In the JIA Persistent stage patients were characterized especially by 1-Butanol, Methyl-isobutyl-ketone, Glucose, 2-Pentanone which showed RP values ranging from 3.8 to 9. Regarding OTUs, Rikenellaceae group decreasing was significantly associated with the JIA Persistent disease stage (Figure 2d). Moreover, in order to achieve an insight on the relationship between omics fingerprints and clinics, different classification models based on clinical data were also established. However, none of these models were able to discriminate amongst JIA patients and reference CTRLs group but also amongst baseline, inactive and persistent disease stages (*p*-values > 0.05) (data not shown).

On the other hand, the information about the dietary habits and the use of prebiotics, probiotics (Table S1) did not allow to formulate any statistically meaningful hypothesis on the possible relation amongst these three factors and the observed discrimination. However, by inspecting the scores’ plot of the PLS-DA models discussed above, in light of the categorizations described by the three possible confounding factors (diet, prebiotics and probiotics), the distribution of JIA samples appeared not *driven* by any of these three effects (data not shown).

### 3.3. Univariate Analysis

Within the group referring to the JIA Inactive-stage, most of the patients were treated with MTX (14/19) similarly to the JIA Persistent-stage group (12/21), whereas at the JIA Baseline-stage the patients were treated with only NSAID (15/20). In order to evaluate the effects due to the pharmacological treatments, we compared the variables that resulted significant in the discrimination between patients and CTRLs, and between patients treated with either NSAID or MTX and those not treated. The results obtained from the univariate analysis (Mann–Whitney Rank Sum Test) showed no significant differences between patients treated and not, regardless therapy type (Text S1).

## 4. Discussion

The integrated metabolomics and targeted-metagenomics results suggest that the observed GM functional and compositional profiles can be specific for the subjects affected by JIA, rather than for each specific disease stage, and regardless of inflammation status, in agreement with previous results obtained by van Dijkhuizen et al. [3].

High levels of Ruminococcaceae, mainly associated to inactive JIA, were also detected in a polyarticular JIA and enthesitis-related arthritis patients (ERA) compared to CTRLs [11]. Moreover, Forbes et al. [17] revealed *Ruminococcus lactaris* and Lachnospiraceae as component of the microbiota profile in patients affected by rheumatoid arthritis (RA), along with other species such as *Clostridium*, *Gordonibacter*, *Eggerthella*, *Bacteroides dentium*, *Lactobacillus* spp.

The changes in the GM profile in JIA patients at the inactive disease stage resulted to have a higher predictive potentiality with respect to those at baseline and persistent disease stages. However, the high levels of Ethanol and Methyl-isobutyl-ketone had invariable characteristics observable in all PLS-DA models. Ethanol may be the end-product of fermentation of different carbohydrates by GM. Thus, several microbial species belonging to Ruminococcaceae, Lachnospiraceae and Clostridiaceae, as well as Enterobacteriaceae are able to produce ethanol [12]. In addition, the increased abundance of these bacterial families was associated with high levels of Ethanol. Ethanol has also been found in large amounts in non-alcoholic fatty liver disease (NAFLD) patients [18]. Furthermore, peculiar changes observed in patients at baseline and persistent disease stage were linked to other alcohols such as 1-Pentanol and 1-Butanol, detected at high levels, compared to CTRLs. On the contrary, the differences between inactive and persistent disease stage patients were not statistically significant.

The higher level of 1-Pentanol and 1-Butanol were previously found in stools of children with NAFLD compared to CTRLs [19], but without any metabolic similarity between the GM of JIA and NAFLD patients. High levels of alcohols have also been observed in the stools of cystic fibrosis children [13], but this metabolic pattern could not define a specific microbial imbalanced activity in JIA patients. Probably, this imbalance could be related to the diet-linked intestinal environment or to a factor linked to an unspecific pathological event than with a metabolism of specific bacteria.

Zhang et al. [20] reported that after the modification of therapy with antirheumatic drugs, there was a partially improved dysbiosis in RA patients, but also highlighted a clinical amelioration.

Dong et al. [21] showed that anti-rheumatic treatments were linked with the partial reversion of dysbiosis. This suggests that the GM plays a crucial role in disease promotion and clinical course and that the intestinal homeostasis is fundamental for the host’s health. The results showed in the present study, in agreement with the previous data [3] carried out in a broad longitudinal study, evidenced that the GM composition and its metabolic activity were not affected by the disease stage.

These results could represent a characteristic phenotypic aspect of JIA individuals, which could be strongly correlated to geographic origin and diet, perfectly in agreement with that previously reported. In fact, dietary habits may represent one of the main variables contributing to the modulation of GM in terms of composition and metabolism [20]. However, we were not able to provide association between dietary habits and GM profiling in JIA patients due to a lack of clear dietary-induced GM modulation. The data fused suggested that the microbiome differences observed between inactive JIA and CTRLs might be related to an unspecific effect dependent on the therapy. Whether the GM composition in JIA Inactive disease was affected by the underlying disease process or past pharmacological treatments, it could not be determined in the present study. Indeed, a limitation of this study originates by an uneven distribution of the therapeutic interventions in different disease stages, as reported in the Results. In fact, the variance introduced by different treatments of the patient set could explain why we did not observe any significant variation between JIA Baseline and Persistent compared to JIA Inactive stages. Therefore, we could only consider the comparison of variables between a single subset of patients (e.g., MTX- or NSAID-treated patients) and related CTRLs.

## 5. Conclusions

The results supported the hypothesis that GM of JIA patients showed an imbalanced state respect to CTRLs in terms of composition and metabolic functionality. Methyl-isobutyl-ketone and Ethanol levels were higher in all JIA patients as compared to the healthy controls’ group. Furthermore, a predictive significant model discriminating JIA Inactive disease stage from healthy subjects was provided. At this point, it cannot be excluded that differences in GM arose owing to therapeutic interventions. Clearly, future investigations should be aimed at replicating these results in other populations, and in different disease stages, elucidating the potential causal role of GM in the pathogenesis of JIA and investigating the interaction amongst host genetics, microbiome and environmental factors that concur in the development of JIA.

## Figures and Tables

**Table 1:** Clinical characteristics of the patients with JIA and CTRLs.

Subjects	Females	Mean Age (S.D) *	Mean JADAS-71 (S.D)	Mean CRP (mg/L) (S.D)	Mean ESR (mm/hour) (S.D)	Use of NSAIDs	Use of MTX	Use of Biologicals
**JIA Baseline**	17/20 (85%)	6.4 (±4.07)	15.95 (±9.76)	1.38 (±1.61)	24.6 (±20.41)	15/20 (75%)	0/20 (0%)	0/20 (0%)
**JIA Inactive**	14/19 (73.6%)	7.6 (±3.98)	0.34 (±0.70)	0.24 (±0.29)	11.10 (±5.76)	3/19 (15.8%)	14/19 (73.7%)	3/19 (15.8%)
**JIA Persistent**	13/21 (61.9%)	6.9 (±4.42)	7.19 (±2.28)	0.98 (±1.43)	18.43 (±14.15)	7/21 (33.3%)	12/21 (57.1%)	3/21 (14.3%)
**CTRLs**	14/25 (56%)	9.76 (±2.86)	Nda **	Nda	Nda	Nda	Nda	Nda

*: S.D: standard deviation; **: Nda: No data associated.

**Table 2:** PLS-DA models in DCV of volatile organic compounds (VOCs) data matrix.

Groups	CCR* 1 (%JIA)	CCR 2 (%Control)	CCR 3 Average (%Total)
CTRL vs. JIA ALL	48.6 ± 3.7	99.22 ± 2.0	62.9 ± 2.7
CTRL vs. JIA Baseline	55.9 ± 4.8	98.3 ± 2.3	78.5 ± 2.6
CTRL vs. JIA Inactive	54.2 ± 8.3	93.1 ± 3.7	75.9 ± 4.9
CTRL vs. JIA Persistent	55.4 ± 4.4	96.8 ± 3.0	77.5 ± 3.2

* Correct Classification Rate: CCR.

**Table 3:** Metabolites derived from PLSA-DA models in DCV of VOCs data matrix.

CTRL vs. JIA All	CTRL vs. JIA Baseline	CTRL vs. JIA Inactive	CTRL vs. JIA Persistent
Methyl-Isobutyl Ketone	1-Pentanol	Methyl-isobutyl ketone	Methyl-isobutyl ketone
1-Butanol	Methyl-isobutyl ketone	1-Hexanol	1-Butanol
Ethanol	1-Butanol	Ethanol	2-Pentanone
Benzaldehyde	2,6-Dimethyl 4 heptanone	2,6-Dimethyl 4 heptanone	1-Pentanol
2-Octanone	1-Hexanol	2-Octanone	Ethanol
2-Nonanone	Acetic Acid	1H-indole	4-Methyl phenol
6-Methyl-5-hepten-2-one	Ethanol	Benzaldehyde	Acetone
1H-indole	Phenol	Phenol	2-Butanone
2-Pentanone	4-Methyl phenol	Acetic Acid	2-Octanone
1-Hexanol	Benzaldehyde	6-Methyl-5-hepten-2-one	Benzaldehyde

Legend: In white and grey boxes are reported lower and higher variables detected in all patients and disease stages’, respectively, compared to CTRLs, respectively. Only values of significant RP (*p* < 0.05) were reported.

**Table 4:** PLS-DA models in DCV of operational taxonomic units (OTUs) at family level.

Groups	CCR * 1 (%JIA)	CCR 2 (%Control)	CCR 3 Average (%Total)
CTRL vs. JIA ALL	60.9 ± 4.2	61.2 ± 6.0	61.0 ± 3.4
CTRL vs. JIA Baseline	48.7 ± 7.1	57.4 ± 5.1	53.3 ± 4.2
CTRL vs. JIA Inactive	67.2 ± 5.1	71.1 ± 5.7	69.4 ± 4.0
CTRL vs. JIA Persistent	58.4 ± 6.2	57.8 ± 6.6	58.1 ± 4.7

* Correct Classification Rate: CCR.

**Table 5:** Microbial features derived from PLS-DA models in DCV of OTUs at family level.

CTRL vs. JIA All	CTRL vs. JIA Baseline	CTRL vs. JIA Inactive	CTRL vs. JIA Persistent
Coriobacteriaceae	Clostridiaceae	Ruminococcaceae	Rikenellaceae
Rikenellaceae	Coriobacteriaceae	Lachnospiraceae	Coriobacteriaceae
Clostridiaceae	Streptococcaceae	Clostridiaceae	Others
Lachnospiraceae	Enterobacteriaceae	Veillonellaceae	Enterobacteriaceae
Enterobacteriaceae	Rikenellaceae	Erysipelotrichaceae	Clostridiaceae
Verrucomicrobiaceae	Peptostreptococcaceae	Coriobacteriaceae	Peptostreptococcaceae
Veillonellaceae	Others	Porphyromonadaceae	Mogibacteriaceae
Streptococcaceae	Alcaligenaceae	Enterobacteriaceae	Lachnospiraceae
Ruminococcaceae	Mogibacteriaceae	Streptococcaceae	Porphyromonadaceae
Erysipelotrichaceae	Lachnospiraceae	Others	Veillonellaceae

Legend: In white and grey boxes are reported higher and lower variables detected in patients compared to CTRLs, respectively. Only values of significant RP (*p* < 0.05) were reported.

**Table 6:** PLS-DA models in DCV of Non-VOCs data matrix.

Groups	CCR * 1 (%JIA)	CCR 2 (%Control)	CCR 3 Average (%Total)
CTRL vs. JIA ALL	54.4 ± 4.2	52.6 ± 7.0	53.9 ± 3.4
CTRL vs. JIA Baseline	57.0 ± 7.6	57.5 ± 8.7	57.3 ± 6.5
CTRL vs. JIA Inactive	46.1 ± 8.3	42.4 ± 9.0	44.0 ± 5.3
CTRL vs. JIA Persistent	44.6 ± 7.8	63.2 ± 6.9	54.5 ± 6.8

* Correct Classification Rate: CCR.

**Table 7:** PLS-DA models in DCV of low-level fused data matrix.

Groups	CCR * 1 (%JIA)	CCR 2 (%Control)	CCR 3 Average (%Total)
CTRL vs. JIA ALL	57.6 ± 4.3	77.4 ± 5.2	63.2 ± 3.0
CTRL vs. JIA Baseline	61.7 ± 7.0	80.3 ± 7.1	71.6 ± 4.9
CTRL vs. JIA Inactive	67.4 ± 7.9	77.1 ± 7.0	72.8 ± 5.1
CTRL vs. JIA Persistent	53.0 ± 5.7	81.2 ± 7.3	68.1 ± 4.3

* Correct Classification Rate: CCR.

## Supplementary Materials

The following are available online at https://www.mdpi.com/2076-2607/8/10/1540/s1, Table S1: Dietary habits, prebiotics and probiotics administration; Table S2: GC-SPME raw data; Table S3: NMR raw data; Text S1: Univariate analysis.

## Abbreviations

JIA	Juvenile idiopathic arthritis
GM	Gut microbiome
CTRLs	Controls
OTUs	Operational taxonomic units
SPME/GC-MS	Solid phase microextraction/Gas-chromatography-mass spectrometry
^1^H-NMR	Proton nuclear magnetic resonance spectroscopy
VOCs	Volatile organic compounds
JADAS	Juvenile Arthritis Disease Activity Score
CRP	C- reactive protein level
ESR	Erythrocyte sedimentation rate
NSAIDs	Nonsteroidal anti-inflammatory drugs
MSC	Number of misclassifications
ROC	Receiver operating characteristic
PLS-DA	Partial least squares discriminant analysis
RP	Rank product
CCR	Correct classification rate
DCV	Double cross-validation
ERA	Enthesitis-related arthritis patients
NAFLD	Non-alcoholic fatty liver disease
OPGG	Ospedale Pediatrico Giannina Gaslini
OPBG	Ospedale Pediatrico Bambino Gesù
ILAR	International League Against Rheumatism
