# Supplementary material for: Fused Omics Data Models Reveal Gut Microbiome Signatures Specific of Inactive Stage of Juvenile Idiopathic Arthritis in Pediatric Patients
Source: Microorganisms. 2020 Oct 6;8(10):1540. doi: 10.3390/microorganisms8101540 (PMC7650812; doi:10.3390/microorganisms8101540)
Supplement: Supplementary file 1 [file microorganisms-08-01540-s001.zip › supplementary material_R1/Supplementary Table S1 Diet Pro Pre.docx]

**Supplementary Table S1.**

Schematic representation related to patients dietary habits (omnivore/carnivorous) and to the pre-probiotics administration (**√** = yes; **̶** = not).

| **Patients** | **Diet** | **Prebiotics use** | **Probiotics use** |
| --- | --- | --- | --- |
| MD_A_1 | omnivore | - | - |
| MD_A_13 | omnivore | - | - |
| MD_A_12 | omnivore | - | - |
| MD_A_34 | omnivore | - | - |
| MD_A_40 | omnivore | - | - |
| MD_A_14 | omnivore | - | - |
| MD_A_41 | omnivore | - | - |
| MD_A_16 | omnivore | - | - |
| MD_A_42 | omnivore | - | - |
| MD_A_19 | omnivore | - | - |
| MD_A_56 | omnivore | - | - |
| MD_A_26 | omnivore | - | - |
| MD_A_49 | omnivore | - | - |
| MD_A_30 | omnivore | - | - |
| MD_A_36 | omnivore | - | - |
| MD_A_57 | omnivore | - | - |
| MD_A_32 | omnivore | - | - |
| MD_A_37 | omnivore | - | - |
| MD_A_58 | omnivore | - | - |
| MD_A_70 | omnivore | - | - |
| MD_A_96 | omnivore | - | - |
| MD_A_71 | omnivore | - | - |
| MD_A_74 | omnivore | - | - |
| MD_A_105 | omnivore | - | - |
| MD_A_77 | omnivore | - | - |
| MD_A_102 | omnivore | - | - |
| MD_A_81 | carnivorous | - | √ |
| MD_A_86 | omnivore | - | - |
| MD_A_97 | omnivore | - | - |
| MD_A_9 | omnivore | - | - |
| MD_A_10 | omnivore | - | - |
| MD_A_15 | omnivore | - | - |
| MD_A_21 | omnivore | √ | √ |
| MD_A_28 | omnivore | - | - |
| MD_A_35 | omnivore | - | - |
| MD_A_43 | omnivore | - | - |
| MD_A_51 | omnivore | - | - |
| MD_A_53 | omnivore | - | - |
| MD_A_61 | omnivore | - | - |
| MD_A_66 | omnivore | - | - |
| MD_A_67 | omnivore | - | - |
| MD_A_3 | omnivore | - | - |
| MD_A_72 | omnivore | - | - |
| MD_A_76 | omnivore | - | - |
| MD_A_85 | omnivore | - | - |
| MD_A_101 | omnivore | - | - |
| MD_A_109 | omnivore | - | - |
| MDP-IGG-S1 | omnivore | - | - |
| MDP-IGG-S2 | omnivore | - | - |
| MDP-IGG-S7 | carnivorous | - | - |
| MDP-IGG-S37 | omnivore | - | - |
| MDP-IGG-S32 | omnivore | √ | - |
| MDP-IGG-S55 | carnivorous | - | - |
| MDP-IGG-S45 | omnivore | - | √ |
| MDP-IGG-S56 | omnivore | - | - |
| MDP-IGG-S9 | omnivore | √ | - |
| MDP-IGG-S8 | carnivorous | - | √ |
| MDP-IGG-S19 | carnivorous | - | √ |
| MDP-IGG-S22 | omnivore | √ | - |
| MDP-IGG-S27 | omnivore | - | - |
| MDP-IGG-S28 | omnivore | √ | √ |
