# Supplementary material for: Fused Omics Data Models Reveal Gut Microbiome Signatures Specific of Inactive Stage of Juvenile Idiopathic Arthritis in Pediatric Patients
Source: Microorganisms. 2020 Oct 6;8(10):1540. doi: 10.3390/microorganisms8101540 (PMC7650812; doi:10.3390/microorganisms8101540)
Supplement: Supplementary file 1 [file microorganisms-08-01540-s001.zip › supplementary material_R1/Supplementary Material Text S1.doc]

**Supplementary Text S1**

The univariate analysis (Mann-Whitney Rank Sum Test) showed a significant statistical difference (p<0,001) consisting in a higher content in Methyl Isobutyl Keton in patients treated with NSAID, compared with the controls.

The same analysis runned on patients who have not been treated with NSAID showed a significant statistical difference in the Ethanol (p=0,012) and Methyl Isobutyl Keton (p<0,001) content, which resulted higher in patients compared with the controls for both of the two metabolites.

The univariate analysis did not show a significant statistical difference (p=0,116) in patients treated with MTX, compared with the controls.

The same analysis runned on patients who have not been treated with MTX compared with the controls, did not show a significant statistical difference (p=0,675).
